# Bearing Strength of Crumb Rubber Concrete under Partial Area Loading

**DOI:** 10.3390/ma13112446

**Published:** 2020-05-27

**Authors:** Xiaoqing Xu, Zhigang Zhang, Yangao Hu, Xin Wang

**Affiliations:** 1 School of Civil Engineering, Chongqing University, Chongqing 400045, China; xq.xu@cqu.edu.cn (X.X.); Wangxin0310apply@163.com (X.W.); 2 Key Laboratory of New Technology for Construction of Cities in Mountain Area (Chongqing University), Ministry of Education, Chongqing 400045, China

**Keywords:** bearing strength, crumb rubber concrete, rubber content, partially loaded, constitutive model

## Abstract

The application of waste tire rubber as aggregates in concrete can help to reduce carbon emissions and achieve green gross domestic product (GDP). However, civil engineers still have concerns about using rubberized concrete in structural members. For the safety of structures, the bearing strength of concrete is a very important parameter to be considered in the design. This paper presented the first experimental and numerical study on the bearing strength of crumb rubber concrete. Prisms of both normal concrete and crumb rubber concrete were tested with loading plates of varying sizes. The test results show that the failure modes and deformation behavior of crumb rubber concrete specimens with different rubber contents were similar to those of normal concrete, and the bearing strength of crumb rubber concrete can be well predicted by current standards for normal concrete. Finite element analysis was performed to further determine the effect of rubber content on the bearing strength of concrete. Proper parameter values for modeling crumb rubber concrete by the concrete damaged plasticity model were investigated. Through the numerical analysis, the reason the rubber content does not have an important effect on the bearing strength of crumb rubber concrete with similar compressive strength was found to be that the influence of rubber content on the tri-axial compression behavior of concrete and the ratio of concrete tensile strength to compressive strength is small. The experimental and numerical results presented in this study provide the insights needed to guide the design of structures utilizing crumb rubber concrete.

## 1. Introduction

The development of the automobile industry is accompanied by the production of a large amount of waste tires. The waste tires are solid wastes that contain up to 90% of vulcanized rubber [1], which cannot be easily decomposed or weathered. Most of the tire rubber is burnt as fuel, which is a process causing poisonous gases, and produces an amount of CO_2_ emission [2], as a result, burdening the environment. Therefore, the reuse and recycling of the tire rubber in an environmentally friendly way is a hot research issue. In the early 1990s, researchers started to use the recycled rubber particles as aggregates in concrete and studied the applicability of this type of concrete for potential structural applications [3,4,5]. Now, it is believed that the development and application of rubberized concrete is beneficial to the diversified development of concrete material and is of great significance for reducing carbon emissions and achieving green gross domestic product (GDP) [6,7,8].

Rubber is a hyper-elastic incompressible material and can withstand large deformation. Rubber does not participate in the hydration reaction in concrete, and thus does not change the chemical properties of concrete. The elastic modulus of rubber is much smaller than the other types of mineral aggregate. Therefore, the rubber particles in concrete act as elastic holes and improve its internal physical structure [9,10]. The effect of rubber particles on the mechanical properties of concrete has been extensively studied in the past decades. A consensus reached by researchers is that the incorporation of rubber particles in concrete reduced its compressive strength and elastic modulus, while improving its hardness and ductility as well as dynamic properties. Rubberized concrete was initially used as pavement and exhibited good performance [7,11,12]. Moreover, rubberized concrete was suggested to be suitable for structural applications owing to its light unit weight and relatively high toughness [3,7,13]. Up to now, the performance of rubberized concrete beams, columns, and beam–column elements has been investigated. However, as Strukar et al. [7] pointed out, the detailed investigations on the use of rubberized concrete for structural applications are still very limited, and there are many problems needed to be solved for promoting its application.

**Figure 1:**
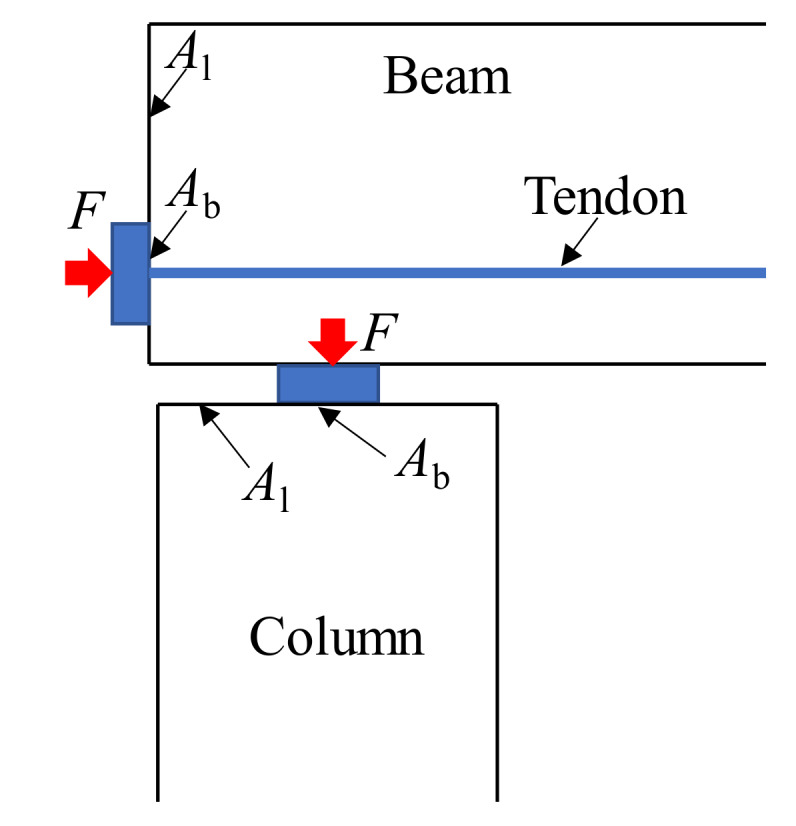
Engineering example for partially-loaded concrete members.

The bearing strength of rubberized concrete is one of the most important issues to be studied. The bearing strength is the strength of a concrete block when the load applied within a small area on top surface of the block, that is, being partially loaded, as shown in Figure 1. Proper estimation of this kind of strength is necessary for the design of load-bearing structures such as concrete hinges, post-tensioned concrete members, and partially-loaded columns or foundations [14]. In previous studies, the flexural stiffness and cracking load of reinforced rubberized concrete beams decreased as the rubber content in concrete increased [15,16,17]. If the prestressing tendons are adopted to eliminate the adverse effect by the addition of rubber on the structural performance of the rubberized concrete beam, the bearing strength of the concrete in the anchorage area of the tendons must be considered as most quality accidents of prestressed concrete structures are caused by insufficient bearing strength. As another prospective application of rubberized concrete in structural members, steel tubes filled with rubberized concrete, which take advantage of the improved ductility and energy dissipation capacities of rubberized concrete as compared with that of normal concrete (NC), present higher ductility than those filled with NC [18]. The tube is often partially loaded as it is used as a bridge pier or a column in high-rise buildings [19]. Therefore, it is important to investigate the bearing strength of rubberized concrete for ensuring the safety of the structures using rubberized concrete. However, there is no report on studies focused on this topic.

The bearing strength of concrete is closely related to its tri-axial mechanical properties [20]. Unfortunately, the tri-axial mechanical properties of rubberized concrete are rarely studied. The only relevant research was conducted by Gholampour et al. [21], who tested cylinder specimens of concrete with rubber replacement ratios of 0%, 6%, 12%, and 18% under active confining pressures up to 25 MPa. The results indicated that the rubber content significantly affects the compressive behavior of actively confined concrete. Under the same confining pressure, an increase in the rubber content results in a decrease of the peak and residual axial stresses. As a reasonable guess, the addition of rubber particles in concrete may also have an impact on the bearing strength of concrete.

In view of the literatures above, it is necessary to carry out experimental research to investigate the bearing strength of rubberized concrete. In addition to the experimental research, numerical simulations can be an effective research method. However, there are limited studies on the simulation of rubberized concrete. Yan et al. [22] and Han et al. [23] conducted numerical analysis on steel and concrete composite structures with rubberized concrete, while Duarte et al. [18] simulated steel tubes filled with rubberized concrete. It was shown by these studies that the concrete damage plasticity (CDP) model in ABAQUS [24] was able to model the strength and failure of rubberized concrete [25,26]. However, previous numerical assessments focused mainly on the performance of structural members, while detailed numerical assessments dealing with the material properties of rubberized concrete are still lacking.

For rubberized concrete, three types of waste tire rubber categorized by particle size were normally used, which are coarse tire chips, fine crumb rubber, and rubber powder, respectively. This paper presents the first experimental and numerical study on the bearing strength of crumb rubber concrete (CRC), in which the crumb rubber was used to replace sand. Prism specimens of NC and CRCs with rubber replacement ratios of 5.0% and 10.1% were loaded as the bearing plates with various sizes. The results of bearing strength were then compared with the existing equations. Finally, in order to examine the bearing strength of CRC with a wide range of rubber replacement ratios and compressive strengths, simulations and parametric assessments were conducted by establishing three-dimensional nonlinear finite element models in conjunction with the concrete damage plasticity model for simulating CRC.

## 2. Bearing Strength of Concrete

**Figure 2:**
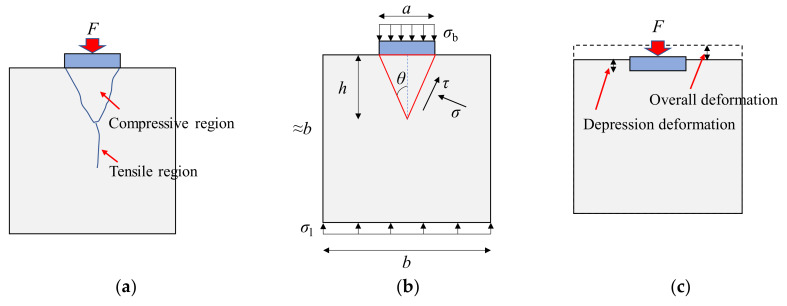
Partially-loaded concrete block: (**a**) failure mode; (**b**) variable description; (**c**) deformation.

On the basis of elastic stress analysis of the partially-loaded concrete block [20], it is known that the stress *σ*_b_ (*F*/*A*_b_), which is concentrated under the loading plate, gradually spreads down to a uniformly distributed stress *σ*_l_ (*F*/*A*_l_), as shown in Figure 2. Part of the concrete under the loading plate is under tri-axial compression, while there is a region below this compressive region where the transverse tensile stress is large [20]. The results of a large number of tests show that the bearing strength of concrete is determined by the failure of these two regions [14,20,27,28]. When the compressive region fails, *σ*_b_ can reach several times the uni-axial concrete compressive strength owing to the confinement provided by the surrounding concrete. According to the tri-axial failure criterion of concrete, the state of confining stresses determines the failure stress of the compressive region. The greater the confining stresses, the higher the failure stress. The tensile region fails when the tensile stress reaches the tri-axial tensile strength of concrete, which attributes to that the tensile region is under tri-axial tension. It is worth noting that the tensile strength of concrete should be less than its uniaxial tensile strength.

The bearing strength of concrete is highly affected by the loaded area, geometry of the loaded members, loading conditions, and its compressive strength [29,30]. Hawkins proposed a classical model to analyze the bearing strength of concrete [31]. It was assumed that the compressive failure is the result of sliding along planes inclined to the direction of principal stress, as a result, a wedge of concrete punches down, and the stress (*σ*) on the surface of the wedge satisfies the following Equation (1).

(1) τ=C+σtanφ

where *τ* and *σ* are the shear stress and normal stress on the wedge surface, respectively; and *C* and *φ* are the cohesion and angle of internal friction of concrete, respectively. On the basis of this failure criterion, a bearing strength equation for concentrically loaded specimens was proposed as follows.

(2) $$f_{\mathrm{cb}}=f_{c}+f_{\mathrm{ct}}\left(\sqrt{\frac{A_{l}}{A_{b}}}-1\right)\cot^{2}\theta$$

where *f*_cb_ is bearing strength of concrete; *f*_c_ is the compressive strength of concrete; *f*_ct_ is the tensile strength of concrete; *A*_l_ is the block gross area; *A*_b_ is the loaded area; and *θ* is the half of the apex angle of the pyramid, which is (45° − *φ*/2), as shown in Figure 2. The formula successfully predicts the key parameters affecting the bearing strength of concrete, including tensile strength and compressive strength, *φ*, as well as load concentration ratio (*A*_b_/*A*_l_). In most current standards (e.g., Eurocode 2 [32], ACI 318 [33]), the design equations for the bearing strength of concrete take the load concentration ratio as the main parameter following the “square root equation” proposed by Hawkins [31], which can be expressed as Equation (3).

(3) $$\frac{f_{\mathrm{cb}}}{f_{c}}=\sqrt{\frac{A_{l}}{A_{b}}}$$

## 3. Experiment Description

### 3.1. Test Specimens

In this study, prisms specimens with dimensions of 150 mm × 150 mm × 300 mm were used. There are four groups as shown in Table 1. The first group used NC, while the other groups used CRC. In order to study the development of bearing strength with time, the CRC5E group, which had the same mix proportion with CRC5, was tested at 14 curing days, while the other specimens were tested at 28 curing days. The mix proportions of concrete are presented in Table 1. The rubber replacement ratio (*ρ*_vr_) refers to the rubber replacement by equal volume of mineral aggregates. As known, the compressive strength of concrete decreases with the increase in rubber content, hence the relatively higher cement quantity was used in CRC to compromise the loss in compressive strength upon the addition of rubber, thus reaching a similar compressive strength as that of NC. Although the usage of cement in CRC cases is relatively higher than that in the NC case, the total CO_2_ emission of CRC cases is still expected to be lower owing to the reuse of waste rubber, which will be discussed later in Section 3.2.

**Figure 3:**
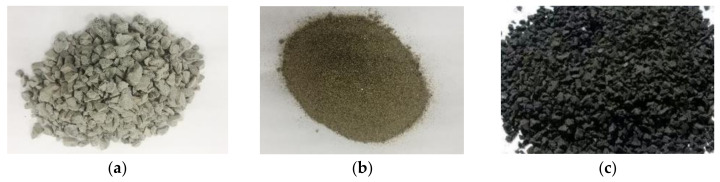
Photos of aggregates: (**a**) stone; (**b**) sand; (**c**) crumb rubber.

Portland cement CEM-42.5 and naphthalene-based water reducer were used. Coarse aggregates were the crushed limestone with particle size between 5 mm and 30 mm. Fine aggregates were medium sand with fineness modulus of 2.5. The crumb rubber has a size range of 2~4 mm and a density of 1.42 × 10^3^ kg/m^3^. The photos of aggregates are presented in Figure 3.

**Figure 4:**
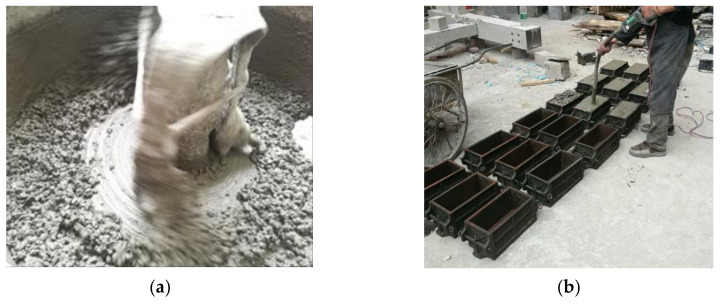
Fabricating specimens: (**a**) concrete mixing; (**b**) concrete casting.

Before casting, the rubber particles were surface-treated with NaOH aqueous solution with 1% mass fraction of NaOH for 30 min, at room temperature, with stirring. Then, the rubber particles were washed with water and dried at ambient temperature. All mineral aggregates, cement, and half of the water were mixed in a mixing machine for 1 min. The rubber particles and the remaining water were then added to the mixer and mixed for another 3 min. Afterwards, the concrete was placed in formworks, and subsequently compacted using a handheld concrete vibrator. Figure 4 presents the photos for the process of fabricating specimens. All specimens were demolded after 24 h, and were then cured under natural outdoor exposure conditions (relative humidity (RH) 70% ± 10% and 15 ± 5 °C) under a shelter until test.

### 3.2. Testing Procedure

**Figure 5:**
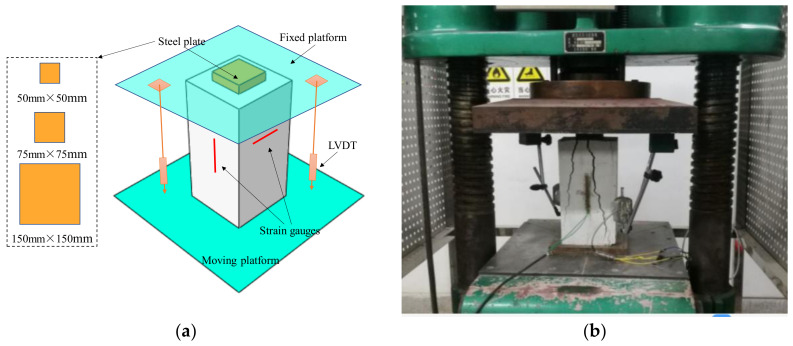
Schematic diagram of partial-contact-loading test setup: (**a**) schematic diagram; (**b**) photo. LVDT, linear variable displacement transformer.

The specimens were tested via a servo hydraulic testing machine with a capacity of 5000 kN. The time and load were recorded by machine readings. In each group, specimens were tested by three square loading plates of 150 mm, 75 mm, and 50 mm sides, respectively. As shown in Figure 5, the steel plate was placed on upper surface of specimens, and the moving platform moved upward to load specimens. Two linear variable displacement transformers (LVDTs) were placed between the fixed platform and moving platform to record the axial deformation. Two strain gauges of 80 mm in length were attached on all specimens, between which one was placed in longitudinal direction at the mid-height of the specimen to measure compressive strain, and the other one was placed in the lateral direction to obtain tensile strain of specimens. During the test, the load was applied in several steps, and the loading speed was controlled under 40 kN/min during each loading step. The load was increased by 40 kN in one loading step within a range of 60% of the predicted ultimate load, and then the load was increased by 20 kN within a range of 60~80% of the predicted ultimate load. When approaching ultimate load, the load was increased by 10 kN or 5 kN. Before conducting each loading step, the load was held for more than 30 s.

## 4. Experimental Results

### 4.1. Fully-Loaded Specimens

The results of compressive strength of prism specimens are listed in Table 2. The mean compressive strength of CRC5 and CRC10 specimens is 21.8 MPa and 20.4 MPa, which is 24.6% and 29.3% lower than that of NC specimens, respectively. The difference in compressive strength between CRC5 and CRC10 specimens was little, indicating that increased cement usage in CRC10 compromised the loss of strength upon increased rubber content. In addition, comparing the results of CRC5E and CRC5 specimens, it can be seen that the 14-day strength of CRC can reach 83.6% of the 28-day strength. According to the FIB design code [34], the ratio was estimated to be 90.2%, indicating that the addition of crumb rubber may have an impact on the development of compressive strength with time.

**Figure 6:**
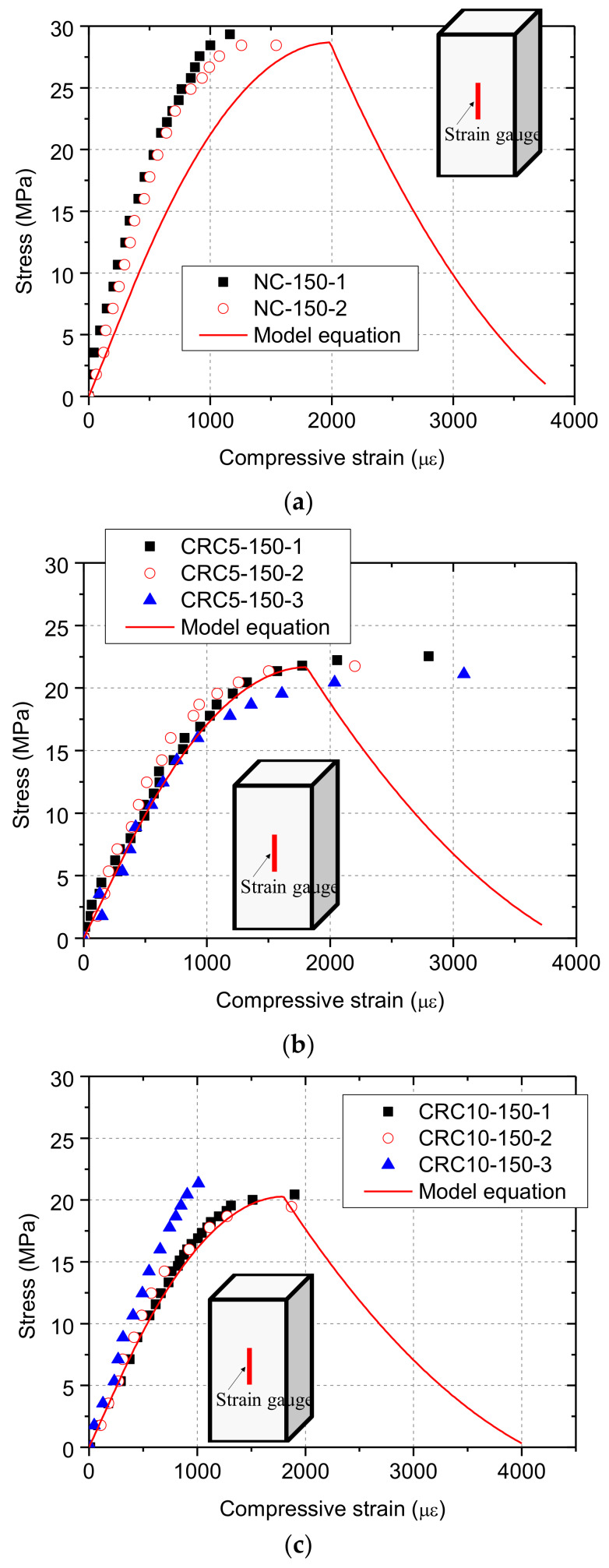
Compressive stress–strain curves of specimens under full contact loading: (**a**) normal concrete (NC)-150; (**b**) crumb rubber concrete (CRC)5-150; (**c**) CRC10-150.

The compressive stress–strain curves of fully-loaded specimens are shown in Figure 6. It is clear that the mean compressive strain of NC when specimens failed was smaller than that of CRC5 and CRC10, among which CRC5 specimen shows the largest strain capacity. The average secant stiffness values between 0 and 0.3 times compressive strength for NC, CRC5, and CRC10 groups are 39.8 GPa, 22.8 GPa, and 21.3 GPa, respectively. It can be seen that CRC specimens had smaller stiffness.

### 4.2. Environment Footprint

**Figure 7:**
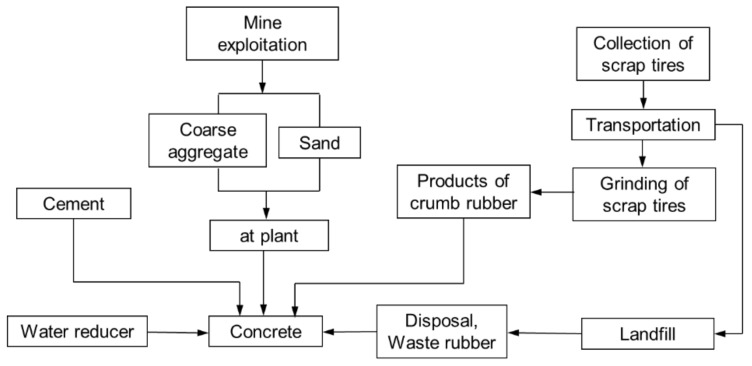
Life cycle assessment (LCA) model of concrete.

In this paper, the impact of addition of rubber into concrete on the environment was also evaluated via the life cycle assessment (LCA) method, which has usually been used as a comparable tool in recent years. Figure 7 presents the LCA model of concrete used in this study. In this model, the total amount of CO_2_ emission of all raw ingredients in concrete, which comes from the whole process of production, delivery, and disposal, was calculated.

During the computations of CO_2_ emissions of concrete, the inventory data of ingredients come from the Chinese life cycle database (CLSD) [35], and are listed in Table 3. Among the ingredients in concrete, as shown in Figure 7, the original sources of coarse aggregate and sand are the exploitation of mine, and then they will be fabricated to the desired size at the plant site. The crumb rubber used in this study comes from the grinding of waste rubber, which produces CO_2_ emission; however, as a waste material, reuse of crumb rubber can avoid the CO_2_ emission during its disposal process, hence it should subtract the CO_2_ emission during disposing waste rubber when conducting the calculation.

**Figure 8:**
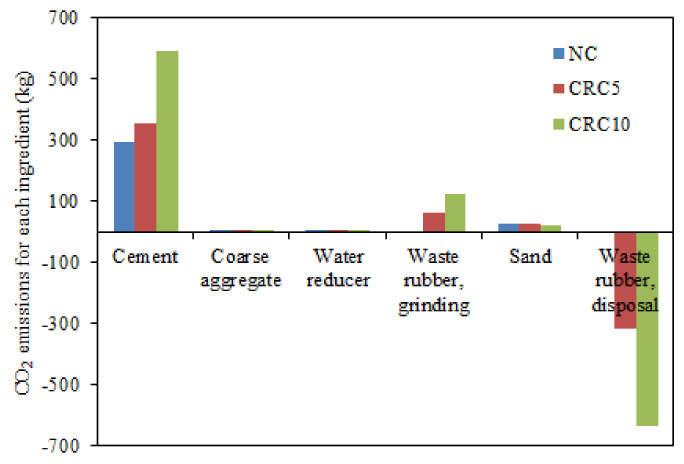
CO_2_ emission for each ingredient in concrete.

Table 4 summarized the calculated results of the amount of CO_2_ emission of concrete. It could be found from Table 4 that the amount of CO_2_ emission decreases as the rubber contents increase. Compared with NC, the reuse of waste rubber in CRC5 and CRC10 significantly reduced CO_2_ emission by 60% and 70%, respectively. This is because that the amount of CO_2_ emission during the process of disposing waste rubber occupies quite a high percentage of concrete, as shown in Figure 8. In summary, the above findings indicate the reuse of rubber in concrete allow it to be more environmentally friendly, albeit with relatively higher cement usage for improving the compressive strength of concrete.

### 4.3. Partially-Loaded Specimens

#### 4.3.1. Failure Modes

**Figure 9:**
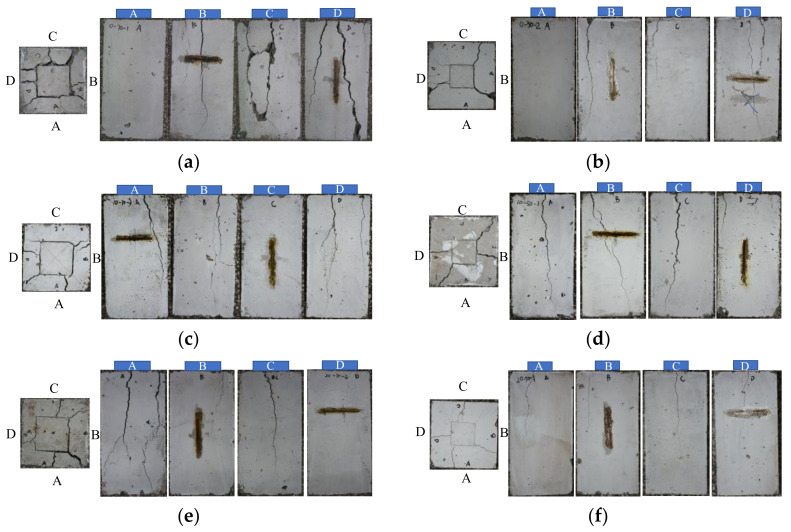
Failure modes: (**a**) NC-75-1; (**b**) NC-50-2; (**c**) CRC5-75-3; (**d**) CRC5-50-1; (**e**) CRC10-75-2; (**f**) CRC10-50-1.

Figure 9 shows the typical failure modes of concrete specimens with different rubber contents. It can be observed that the top surface of concrete under the loading plate presents an obvious subsidence; meanwhile, several cracks spread radially from the loaded area. These cracks propagated vertically from top to bottom of specimens, and the crack width decreased gradually. It can be seen by the naked eye that the crack width was smaller as the specimen was loaded by a smaller loading plate. Although the number and width of cracks on specimens were different, and specimens with different rubber contents exhibit the similar failure mode. In addition, cracks on concrete occurred before the load reached its peak for most specimens, except for only two specimens, NC-50-3 and CRC10-50-3, which cracked and failed almost at the same moment.

**Figure 10:**
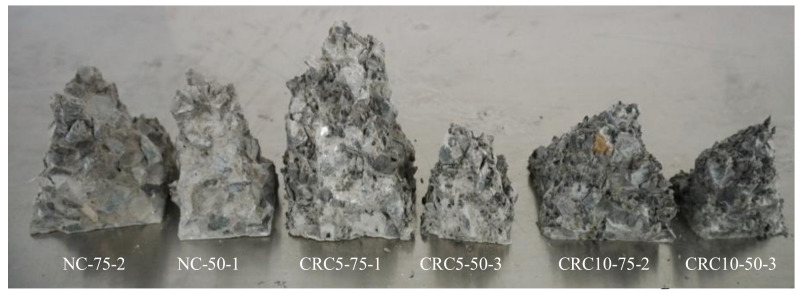
Failure wedges.

After reaching ultimate bearing capacities, the loading was continuously applied to six specimens for the punching out of the concrete wedges, which are shown in Figure 10. Table 5 lists the measured height of failure wedges and calculated angle of internal friction. It is observed that the value of the angle was not much affected by the rubber replacement ratio in concrete and was about 50°, except for that of the CRC10-75-2 specimen.

#### 4.3.2. Strain Results

**Figure 11:**
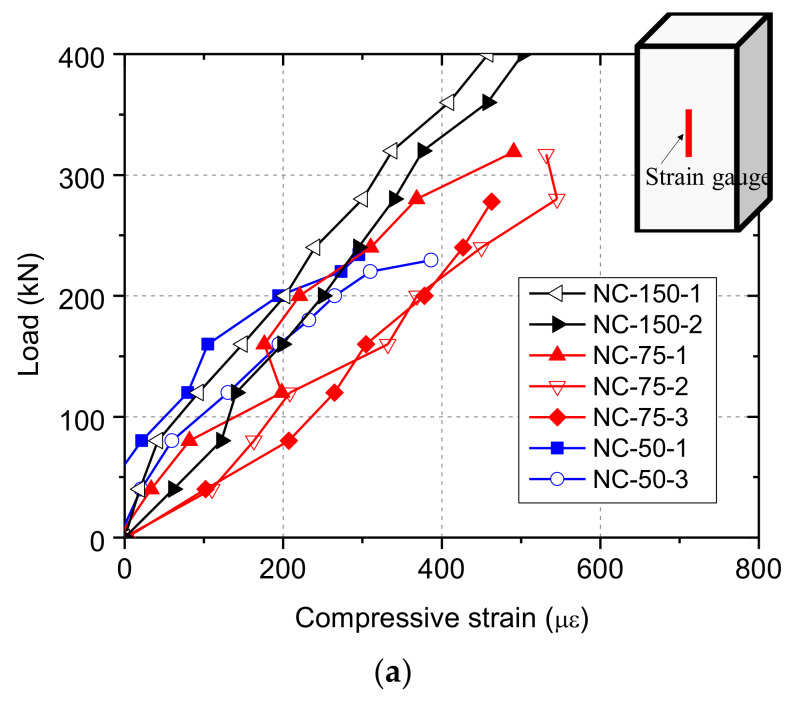

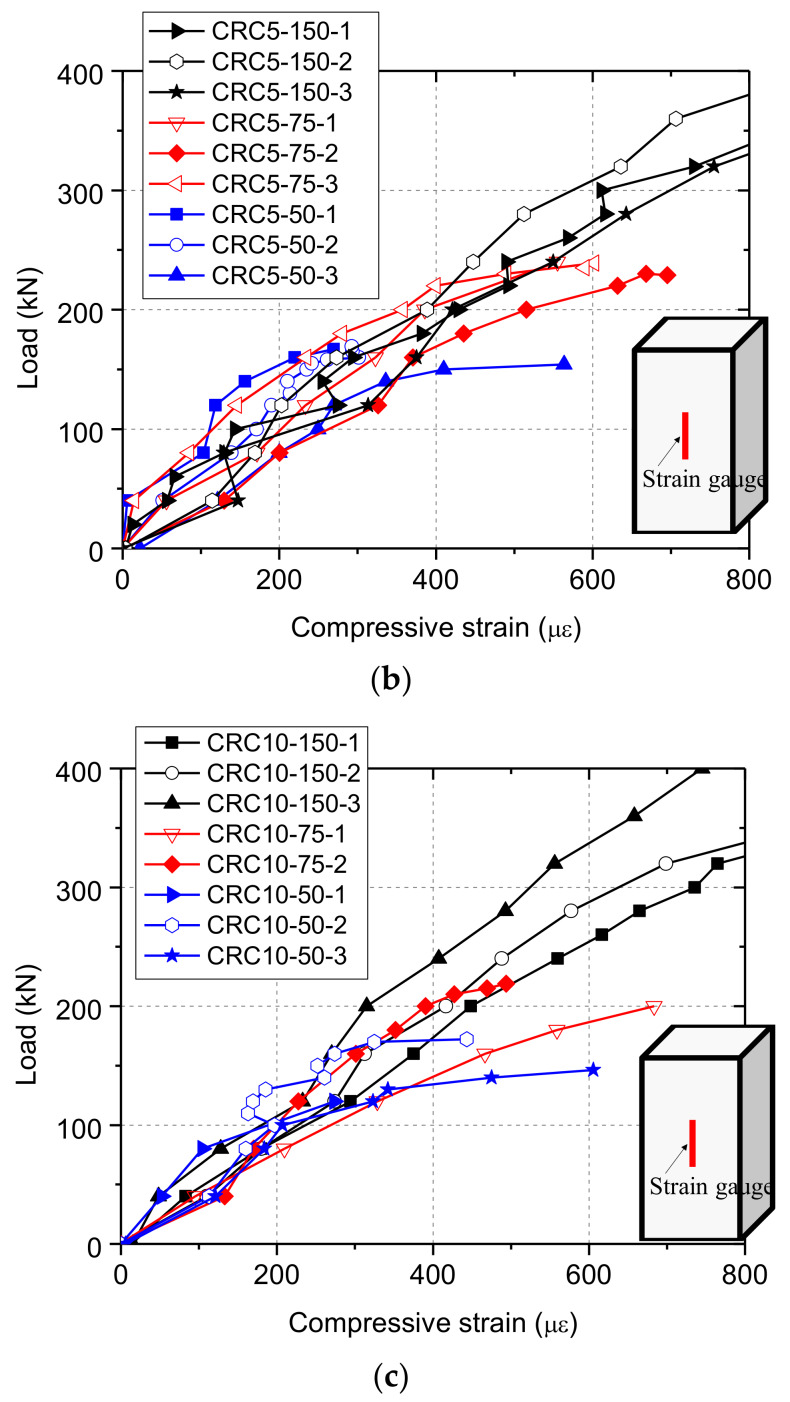
The load–strain curves of specimens under partial contact loading: (**a**) NC; (**b**) CRC5; (**c**) CRC10.

Figure 11 plots the measured load–strain relationship of partially-loaded specimens. In each group, the compressive strain increased with increasing load for partially-loaded specimens, of which the change tendency was basically the same as that for fully-loaded specimens. This indicates that, within the length of the strain gauge, the compressive load was basically borne by the entire cross section of partially-loaded specimens, and the stress had become uniform and close to *σ*_l_, as shown in Figure 2. Therefore, the measured compressive strain could represent overall deformation of the loading contact area on specimens, as shown in Figure 2c. Prior to failure, the compressive strain increased rapidly as the applied load increased, which could be attributed to the formation of cracks on specimens.

**Figure 12:**
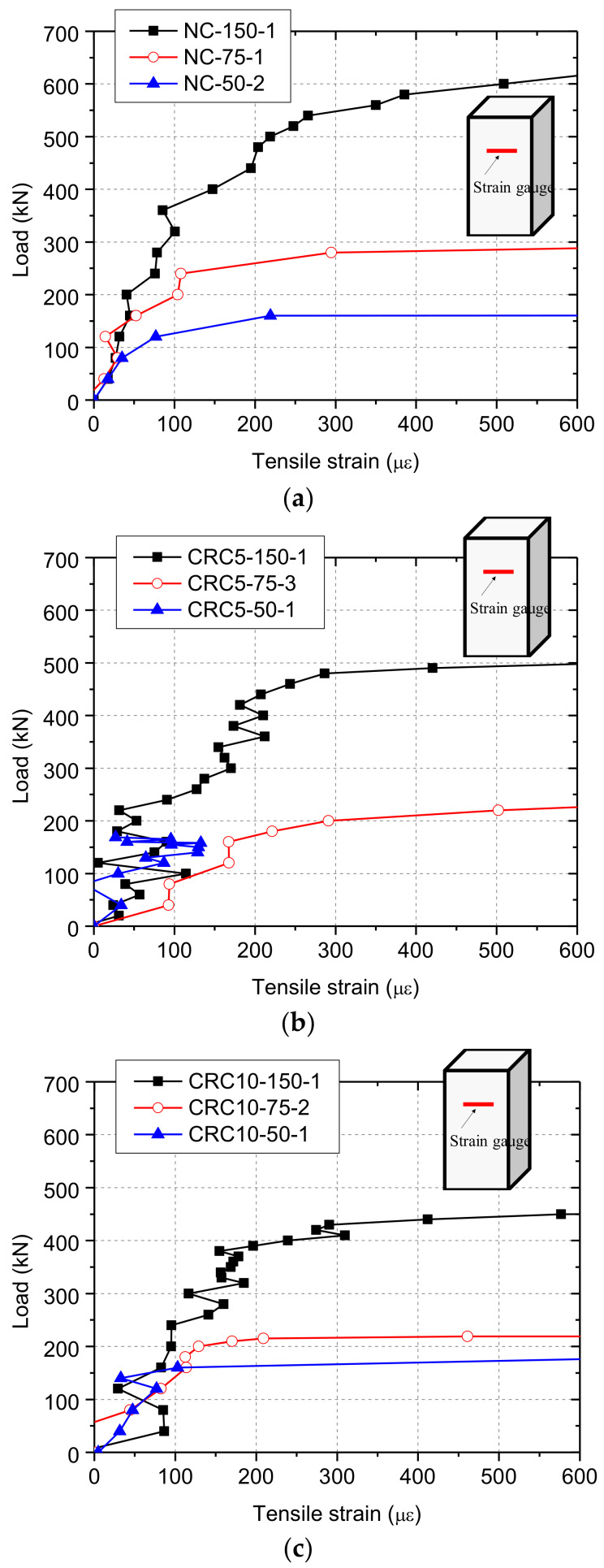
Results of tensile strain: (**a**) NC; (**b**) CRC5; (**c**) CRC10.

Figure 12 shows the typical load–tensile strain relationship of specimens. Obviously, the tensile strain in partially-loaded specimens increased faster than that in the fully-loaded specimens, especially when the load approached its peak. Assuming that the cracking load was the load when the tensile strain reached 200 με and cracks were visible to the naked eye, it was clear that the smaller the loaded area, the closer the cracking load to the ultimate load. In specimens where the loading area was smaller, the confinement to the region under tri-axial compression was larger and more sensitive to the concrete cracks. Thus, in these specimens, there is a tendency that the load reached its peak once cracks developed.

#### 4.3.3. Displacement Results

**Figure 13:**
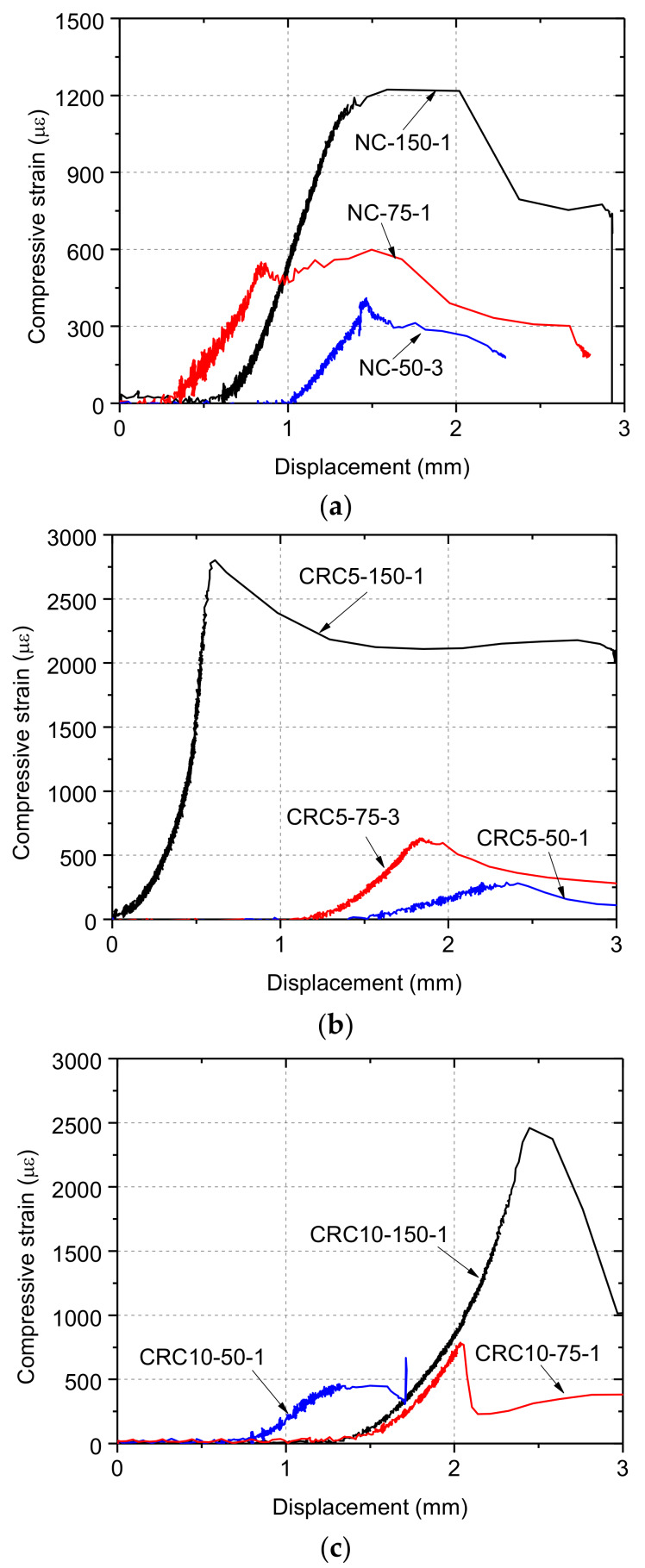
Displacement results: (**a**) NC; (**b**) CRC5; (**c**) CRC10.

The relationship between the measured displacement and compressive strain is shown in Figure 13. The displacement when the compressive strain was zero was caused by the gap between the fixed platform and the specimen. The displacement measured in this paper included both the overall vertical compression of the specimen and depression formed beneath the loaded area, as shown in Figure 2c. As mentioned above, compressive strain represents the overall deformation of specimens. Then, the ratio of measured displacement to compressive strain, that is, the stiffness of curves, would decrease when the depression deformation increases. The curves of specimens under a smaller loading plate generally had smaller stiffness. This means that, the smaller the loaded area, the larger the depression deformation.

#### 4.3.4. Bearing Strength

In this study, only prism specimens concentrically loaded through a rectangle steel plate were used, hence the following equations are provided for simplicity:

(4) $$\beta_{c}=\frac{f_{\mathrm{cb}}}{f_{c}}$$

(5) $$\beta_{L}=\sqrt{\frac{A_{l}}{A_{b}}}=\frac{b}{a}$$

where *β*_c_ is the ratio of bearing strength to compressive strength; *β*_L_ is the square root function of the ratio of supporting area (*A*_l_) to area of the bearing plate (*A*_b_); and *a* and *b* are the side lengths of the loading plate and concrete block, respectively. Then, Equation (3) can be written as

(6) $$\beta_{c}=\beta_{L}$$

**Figure 14:**
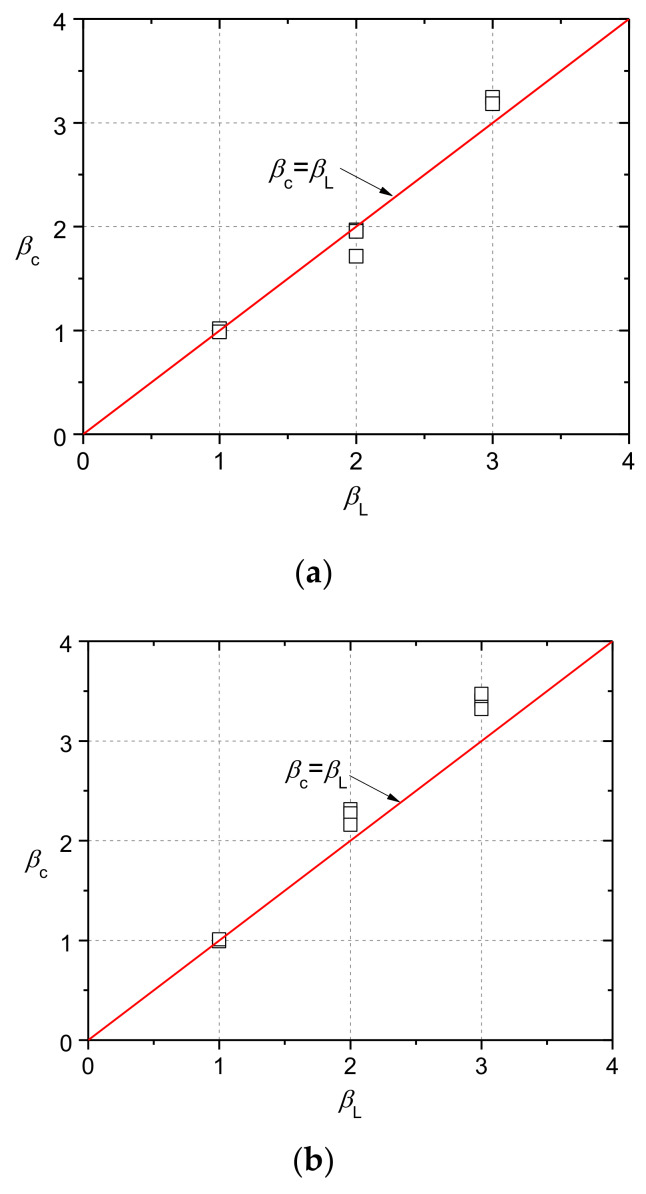

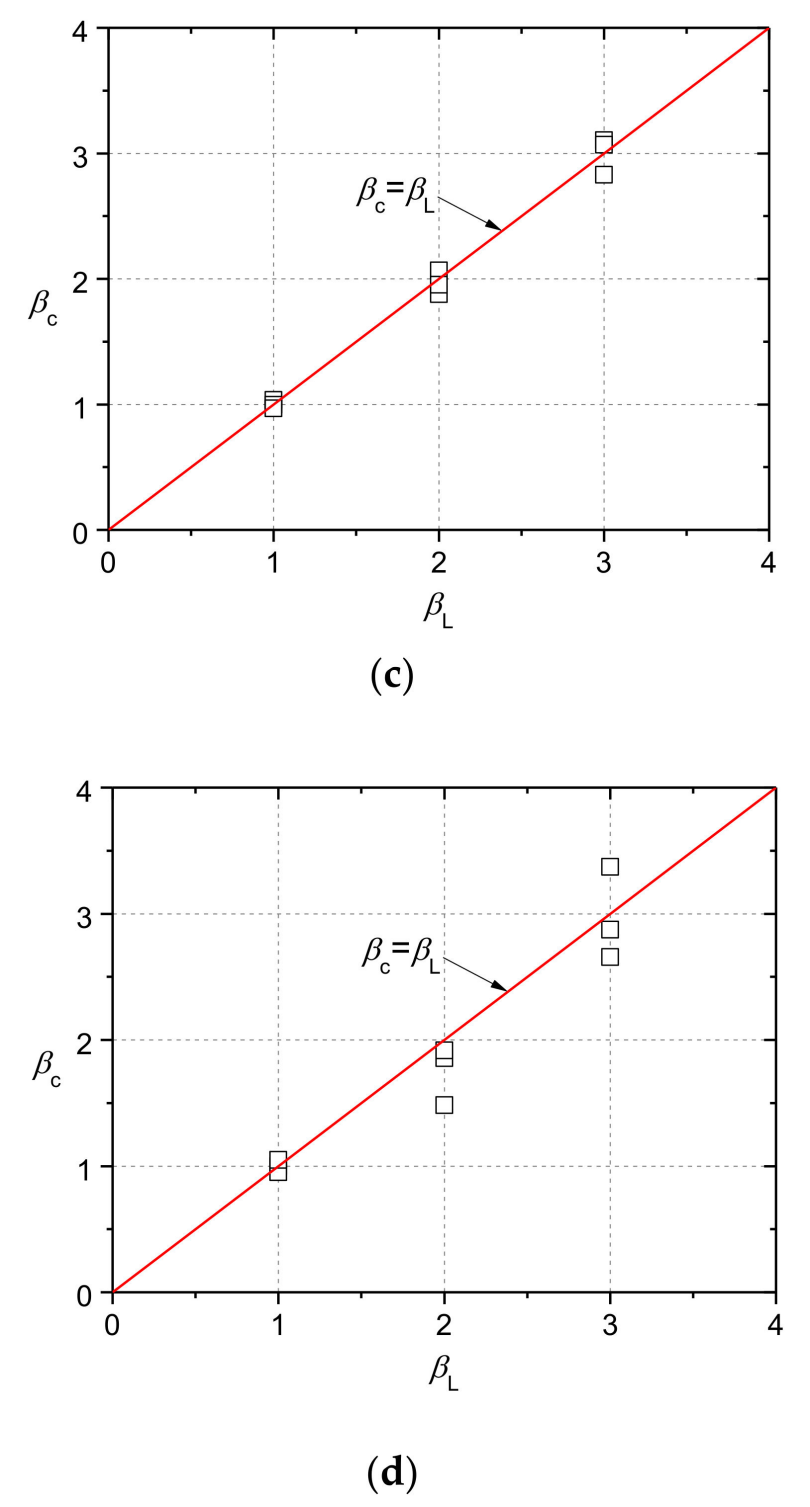
Results of bearing strength: (**a**) NC; (**b**) CRC5E; (**c**) CRC5; (**d**) CRC10.

The bearing strength of the specimen was calculated according to Equation (7), and the results are shown in Table 2.

(7) $$f_{\mathrm{cb}}=\frac{F_{u}}{A_{b}}=\frac{F_{u}}{a^{2}}$$

where *F*_u_ is the ultimate load. The ratio *β*_c_ was calculated. Figure 14 plots the results of each group and compares them with the curve based on Equation (6). *β*_c_ increased almost linearly with *β*_L_, and Equation (6) can give a good prediction. Moreover, there was not much difference between NC and CRC specimens tested at the same curing age in term of *β*_c_. Thus, the current standards for NC could be applied on CRC. Regarding the effect of the curing age, *β*_c_ for CRC with a rubber replacement ratio of 5% at 14 curing days was slightly higher than that at 28 days. Equation (6) gives the conservative estimation for *β*_c_.

Although plenty of studies have shown that rubber content affects the mechanical properties of concrete, the test results of partially-loaded prisms in this paper show that CRC specimens exhibited similar failure mode and deformation behavior to those of NC specimens, and the rubber content had a negligible influence on *β*_c_. The reason was not clear yet, and further study was carried out by the subsequent finite element analysis.

## 5. Finite Element Analysis

The range of parameters studied in the experiment above was small. In view of this limitation of experimental research, finite element models for specimens were established, and the nonlinear constitutive model of rubberized concrete was introduced. The influence of the rubber content on bearing strength of CRC was revealed through a parametric study.

### 5.1. General

**Figure 15:**
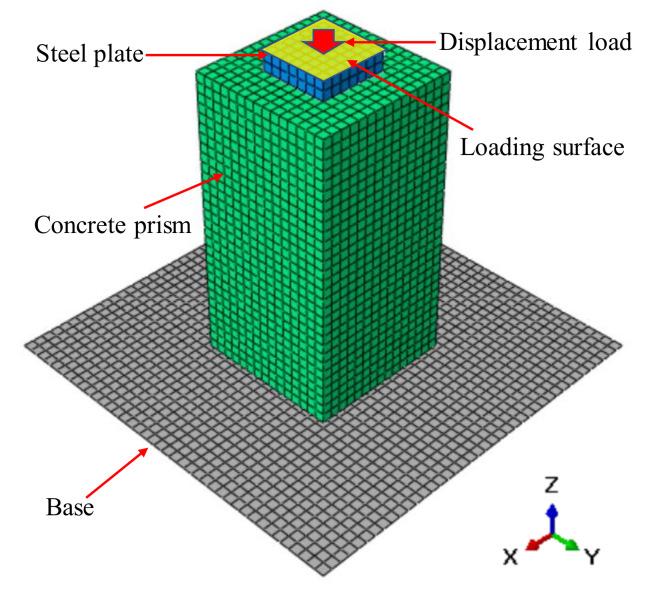
Finite element model.

The finite element commercial package ABAQUS [24] was adopted to establish the models. As shown in Figure 15, the model consisted of the steel plate, concrete prism, and base. The geometry of the model was consistent with the actual dimensions of tested specimens. The surface-to-surface contact was established for all interfaces. The coefficient of friction was assumed to be 0.3, as discussed later. Uniformly distributed displacement load was applied to the upper surface of steel plate, while the reference point of the rigid base was fixed in all directions. C3D8R solid elements were used to model steel plate and concrete prism, and R3D4 elements modeled the rigid base. The mesh size was determined to be 10 mm after a sensitivity study. Generally, the number of the elements was about 6700.

### 5.2. Material Models

The rigid plastic model was used to model steel material. The Young’s modulus was taken as 210 GPa, while the yield strength was taken as 350 MPa.

The concrete damaged plasticity (CDP) model in ABAQUS has been commonly used to model the mechanical behavior of CRC, with its applicability being verified [18,25,26]. The CDP model was also adopted in this study. The yield function, flow rule, hardening rule, and uniaxial stress–strain curves need to be defined for the model. As only monotonic loading was considered herein, the strain–damage relation was not defined.

#### 5.2.1. Yield Function

**Figure 16:**
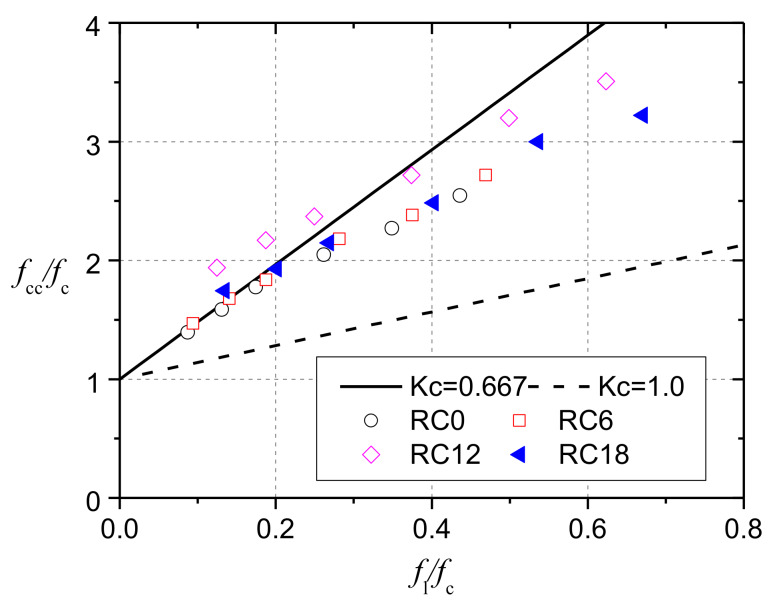
Compressive strength of rubberized concrete under active confinement.

The yield function will affect the ultimate capacity of the model. According the yield function adopted by the CDP model [24], the failure envelope of concrete under tri-axial stress state can be written as

(8) $$\frac{1}{1-\alpha }\left[\sqrt{3J_{2}}+\alpha I_{1}+\beta \langle \sigma_{\max}\rangle -\gamma \langle -\sigma_{\max}\rangle \right]-f_{c}=0$$

where $\langle x\rangle =\left(\left|x\right|+x\right)/2$; *J*_2_ is the second invariant of the stress deviator; *I*_1_ is the first invariant of stress; *σ*_max_ is the algebraically maximum principle stress; and *α*, *β,* and *γ* are dimensionless constants as follows:

(9) $$\alpha =\frac{\left(f_{b0}/f_{c0}\right)-1}{2\left(f_{b0}/f_{c0}\right)-1}$$

(10) $$\beta =\left(1-\alpha \right)\left(f_{c0}/f_{t0}\right)-\left(1+\alpha \right)$$

(11) $$\gamma =\frac{3\left(1-K_{c}\right)}{2K_{c}-1}$$

where *f*_b0_ and *f*_c0_ are the initial equibiaxial and uniaxial compressive yield stresses, respectively; *f*_t0_ is the initial uniaxial tensile yield stress; and *K*_c_ is a constant that determines the shape of yield surface on concrete in the deviatoric plane. The failure wedge of concrete was under tri-axial compression. The compressive meridian when is concrete under tri-axial compression should be paid attention to, which can be derived from Equation (8) as follows:

(12) $$\left(2\gamma +3\right)\sqrt{3J_{2}}+\left(\gamma +3\alpha \right)I_{1}=\left(1-\alpha \right)f_{c}$$

where *f*_b0_ and *K*_c_ should be obtained from equibiaxial and multiaxial compression tests on concrete. Experimental values of (*f*_b0_/*f*_c0_) for NC are in the range of 1.10 and 1.16. A value of *K*_c_ = 0.667 is typical for NC [24]. However, the experimental data on CRC are rare, and the effect of rubber content on these parameters has not been studied yet. Gholampour et al. [21] conducted the first experimental study on the axial compressive behavior of rubberized concrete with rubber replacement ratios of 0%, 6%, 12%, and 18% (RC0, RC6, RC12, and RC18, respectively) under active confinement. The experimental data were plotted in Figure 16, where the abscissa is the ratio of the hydrostatic pressure *f*_l_ to *f*_c_ and the ordinate is the ratio of tri-axial compressive strength *f*_cc_ to *f*_c_. The conclusion was drawn that the axial strength and strain of rubberized concrete increase with confining pressure, following a similar trend to that in NC. This indicates that it is reasonable to use the same parameters as NC for CRC in the yield function.

A value of *K*_c_ = 0.667 was suggested by many researchers when modeling CRC [18,23,25]. Here, the rationality of the value was evaluated by test data reported by Gholampour et al. [21]. Figure 16 plots the curves for Equation (12) when the values of *K*_c_ were taken as 1.0 and 0.667. Under small hydrostatic pressures, the value of 0.667 predicted the test data well, while under high hydrostatic pressures, a larger *K*_c_ value was appropriate. Therefore, it is necessary to choose an appropriate *K*_c_ value for accurately predicting bearing strength.

#### 5.2.2. Flow Rule

A non-associated plastic potential flow rule is used in the CDP model:

(13) $$G=\sqrt{{\left(e\sigma_{t0}\tan\varphi \right)}^{2}+3J_{2}}+\frac{1}{3}\tan\varphi I_{1}$$

where *G* is the flow potential; *e* is a parameter, referred to as the eccentricity; *σ*_t0_ is the uniaxial tensile stress at failure; and *φ* is the dilation angle. The eccentricity parameter was taken as 0.1 for all of the models in this study. The dilation angle varies with the type of concrete and concrete stress states. *φ* was taken as 45° by Han et al. [23] when modeling the headed stud shear connectors in CRC. For the finite element analysis of CRC beam–column members, the sensitivity study carried out by Xu et al. [25] showed that *φ* affected the load capacity and stiffness of the member, and good simulation results were obtained when the value of *φ* = 30° was chosen. Duarte et al. [18] believed that the *φ* value for CRC was lower than that for NC, because of the ability of rubber particles to prevent crack propagation and their incompressible behavior. In their finite element models for short steel tubes filled with rubberized concrete, a lower *φ* value was used for CRC with a higher content of rubber. In view of the existing inconsistency in the *φ* value for CRC, the effect of *φ* on the bearing strength was discussed later.

#### 5.2.3. Uniaxial Stress–Strain Curves

A number of experimental studies on the mechanical properties of rubberized concrete showed that the addition of rubber particles significantly reduced the strength (compressive, tensile, and flexural), elastic modulus of concrete, and ductility [8]. Existing constitutive stress–strain curves for NC were found to be not valid for CRC [36]. This paper used the constitutive model proposed by Bompa et al. [6], who took into account all these effects of rubber particles on the mechanical properties of concrete. Both the compressive and tensile stress–strain curves of rubberized concrete were divided into an ascending part and a descending part. Influences of rubber content and size of rubber particle were considered. The formulas for calculating the main mechanical characteristics of rubberized concrete including the compressive and tensile strength, elastic modulus, and strains at significant levels of stress are as follows:

(14) $$f_{\mathrm{cr}}=\frac{1}{1+2{\left(\frac{3\lambda \rho_{\mathrm{vr}}}{2}\right)}^{3/2}}f_{c0}$$

where *f*_cr_ is the compressive strength of rubberized concrete; *ρ*_vr_ is the rubber replacement ratio; *f*_c0_ is the compressive strength of concrete without rubber content; and *λ* is the function of the replaced mineral aggregate size:

(15) $$\lambda =\left\{2.43\to d_{g}\in \left(0,5\right);\;2.90\to d_{g}\in \left(0,d_{g,\max}\right);\;2.08\to d_{g}\in \left(5,d_{g,\max}\right)\right\}$$

where *d*_g_ is the replaced aggregate size and *d*_g,max_ is the maximum mineral aggregate size.

(16) $$f_{\mathrm{ctr}}=0.26f_{\mathrm{cr}}^{2/3}$$

(17) $$E_{\mathrm{cr}}=12{\left(\frac{f_{\mathrm{cr}}}{10}\right)}^{2/3}$$

where *f*_ctr_ and *E*_cr_ are the tensile strength and elastic modulus of rubberized concrete, respectively.

(18) $$\varepsilon_{\mathrm{cr}1,1}=\left(1-\rho_{\mathrm{vr}}\right)\varepsilon_{\mathrm{cr}0,1}$$

where *ε*_cr1,1_ and *ε*_cr0,1_ are the crushing strain of rubberized concrete and normal concrete, respectively.

(19) $$w_{\max,r}=w_{\max,0}+0.3\rho_{\mathrm{vr}}$$

where *w*_max,r_ and *w*_max,0_ are the maximum crack width of rubberized concrete and normal concrete, respectively. The calculated compressive strain–strain curve for each group is shown in Figure 6, and the main parameters were calculated by adopting the measured compressive strength.

The Poisson’s ratio of the rubber particles is close to 0.5. As a result, the rubberized concrete has a higher Poisson’s ratio (*v*) than NC. Its effect on bearing strength needs to be discussed. Duarte et al. [18] estimated *v* of the rubberized concrete based on the ‘‘rule of mixtures”:

(20) $$v_{\mathrm{RuC}}=v_{\mathrm{NC}}V_{\mathrm{concrete}}+v_{\mathrm{rubber}}V_{\mathrm{rubber}}$$

where *v*_RuC_ and *v*_NC_ are the Poisson’s ratio of rubberized concrete and normal concrete, respectively; *v*_rubber_ is the Poisson’s ratio of rubber particles; and *V*_concrete_ and *V*_rubber_ are the volumetric fraction of concrete matrix and rubber particles in rubberized concrete mixes, respectively. For CRC5 and CRC10 groups in this study, the values of *V*_rubber_ were 7% and 14%, respectively. By assuming that *v*_NC_ equals 0.16, the values of *v*_RuC_ for CRC5 and CRC10 groups were calculated to be 0.18 and 0.21, respectively.

### 5.3. Model Calibration and Verification

The key parameters in the CDP model were calibrated with the test results of NC group specimens. In total, 168 models were established and the influences of these parameters on the calculation results of bearing strength for models with the side lengths of the loading plate increasing from 30 mm in an increment of 20 mm to 150 mm were discussed.

**Figure 17:**
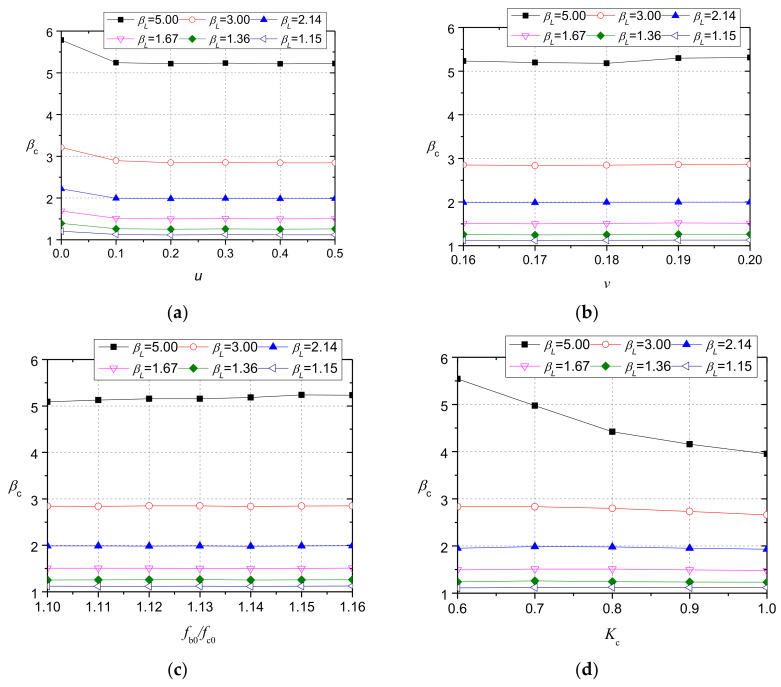

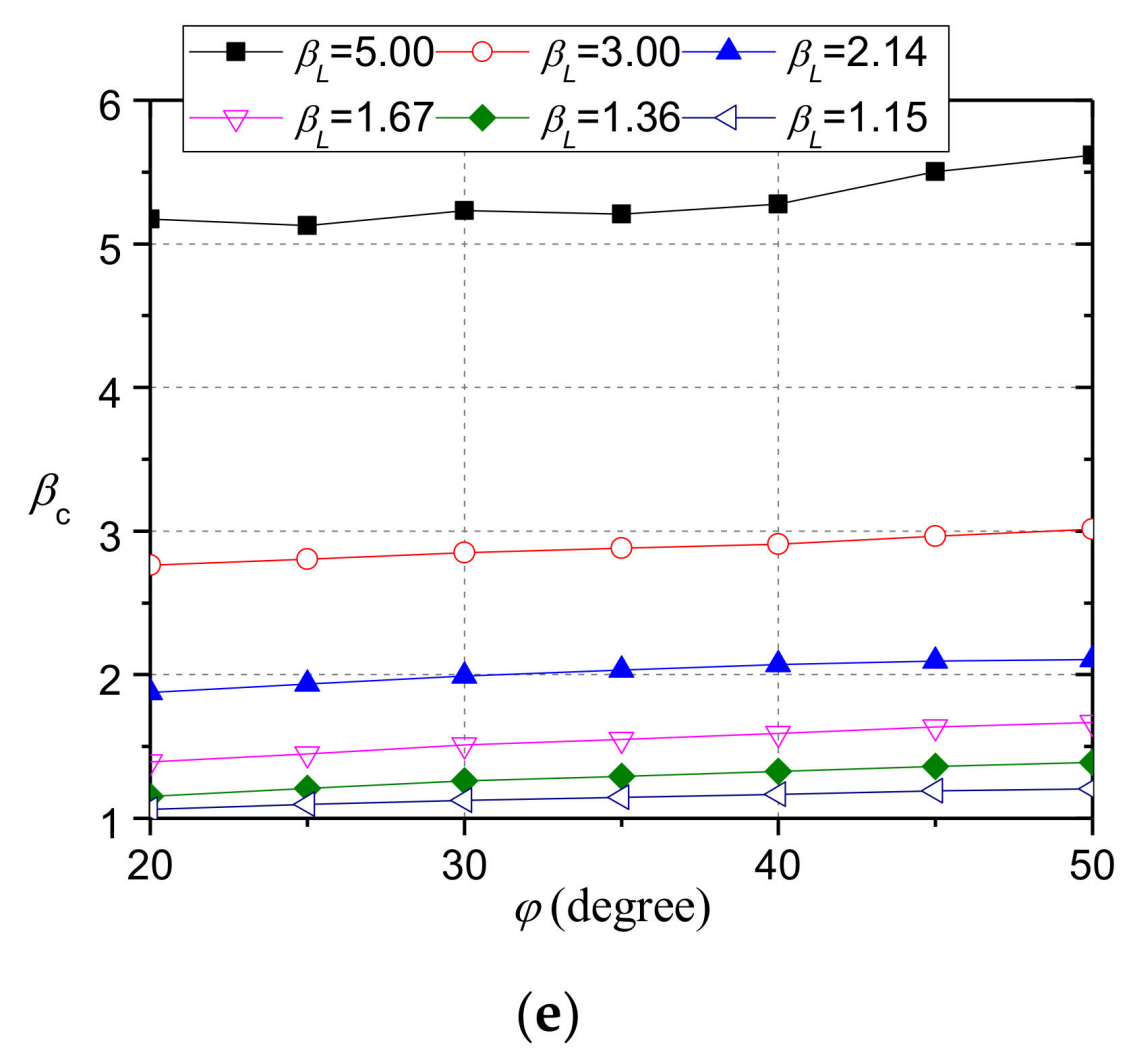
Results of the parametric study: (**a**) *u*; (**b**) *v*; (**c**) *f*_b0_/*f*_c0_; (**d**) *K*_c_; (**e**) *φ*.

These parameters included the coefficient of friction (*u*), *v*, the ratio (*f*_b0_/*f*_c0_), *K*_c_, and *φ*. The initial values of these parameters were taken as their commonly used values, which were 0.3, 0.16, 1.16, 0.667, and 30°, respectively. When one of the parameters was calibrated, the initial values were taken for the other parameters. As shown in Figure 17a, when *u* is greater than 0.1, its influence on the calculation results can be ignored, so the value of 0.3 was used for NC models. As shown in Figure 17b,c, the influences of Poisson’s ratio and (*f*_b0_/*f*_c0_) were also small, so the initial values were taken. The calculation results in Figure 17d show that *β*_c_ decreases with the increase of the value of *K*_c_. The trend is more obvious when *β*_L_ is larger, because the confining stresses in the compressive region increase with *β*_L_, and *K*_c_ has a greater impact on the concrete failure strength when concrete is under a larger confining pressure, as shown in Figure 16. Considering that the maximum value of *β*_L_ in this study was 3.0, the commonly used 0.667 was adopted for simplicity. Figure 17e shows that *β*_c_ increases with the increase of *φ*, which agrees with the trend predicted by Hawkin’s formula [31]. In view of the measured values of *φ* for the failure wedges in NC specimens, *φ* was taken as 50°.

**Figure 18:**
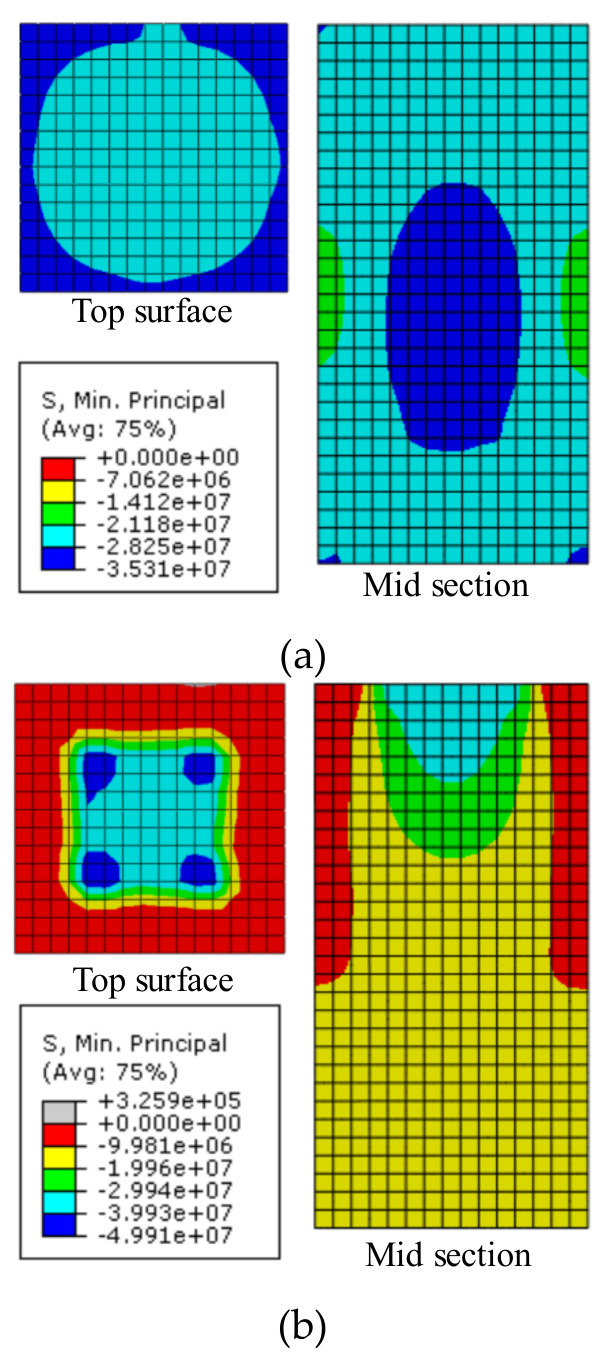

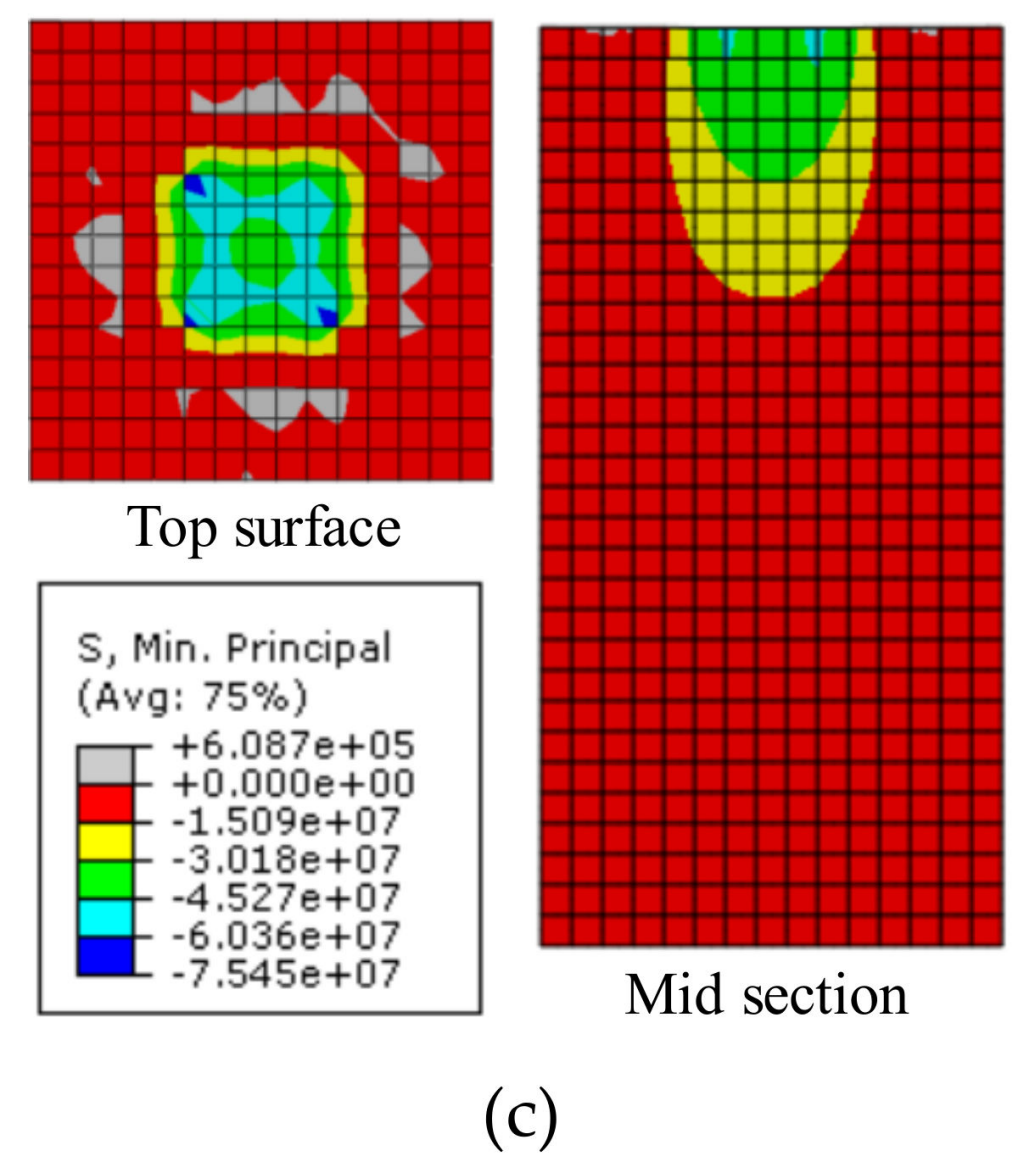
Minimum principal stress (Pa): (**a**) NC-150; (**b**) NC-75; (**c**) NC-50.

After the values of the key parameters were determined, the modeling method was verified by comparing the simulation results with the test results of the NC group. The calculated minimum principal stress state of the specimen at ultimate load was presented in Figure 18. Obvious wedges of concrete under high compressive stress (greater than 80% of the bearing strength) were observed in the models for partially-loaded specimens. At the same time, the size of wedges increased with the size of the loading plate. It can be concluded that the simulated failure mode matched well with the experimental ones.

**Figure 19:**
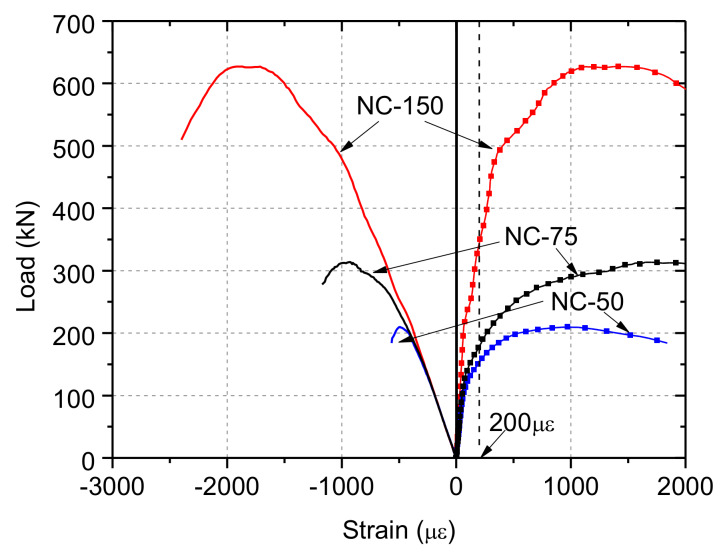
Strain–load curves.

**Figure 20:**
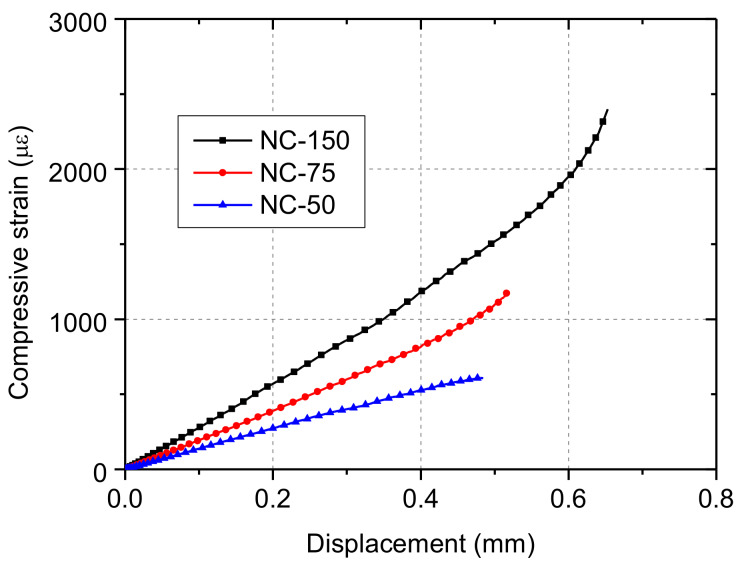
Displacement–compressive strain curves.

Figure 19 shows the calculation results of the average strain within the length of the strain gauge. The finite element models successfully simulated the strain variation observed during the tests. Further, assuming that visible cracks formed when the tensile strain reached 200 με, it can be clearly seen that NC-50 specimen had the smallest ratio of the cracking load to ultimate load, which was consistent with the experimental observations. At the same time, the calculation results of displacement–strain curves shown in Figure 20 clearly illustrate the effect of depression deformation on the overall deformation.

The values of *β*_c_ calculated by the NC-75 and NC-50 models were 2.00 and 3.01, respectively, which were close to the mean values of test results of 1.88 and 3.21, respectively. On the basis of the comparison, it was known that the modeling method can be adopted to analyze the bearing strength of CRC.

### 5.4. Effect of Rubber Content on Bearing Strength

**Figure 21:**
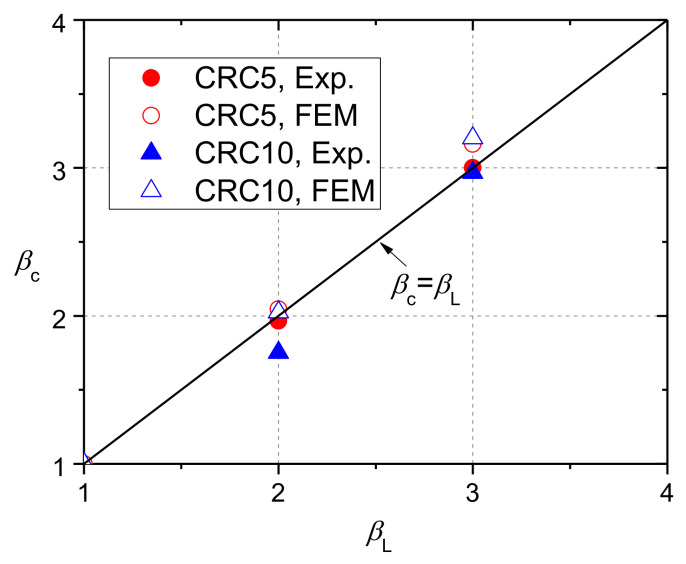
Comparison between the values of *β*_c_ from finite element models (FEM) and experiments.

The values of *u*, (*f*_b0_/*f*_c0_), and *K*_c_ were not changed in finite element models for CRC specimens. Moreover, *v*_RuC_ was taken as the same value as *v*_NC_, because the parametric analysis had shown that it had little effect on bearing strength. In addition, because the measured values of *φ* for CRC specimens were close to that of NC specimens, the value of *φ* was also not modified. It can be seen from Figure 17e that the difference in the calculation results when 30° and 50° were taken for *φ* was within 10%. Figure 21 shows the calculated results for CRC5 and CRC10 groups. It is clear that the rubber content has a negligible effect on the bearing strength, which was consistent with the conclusion obtained from the experimental results.

**Figure 22:**
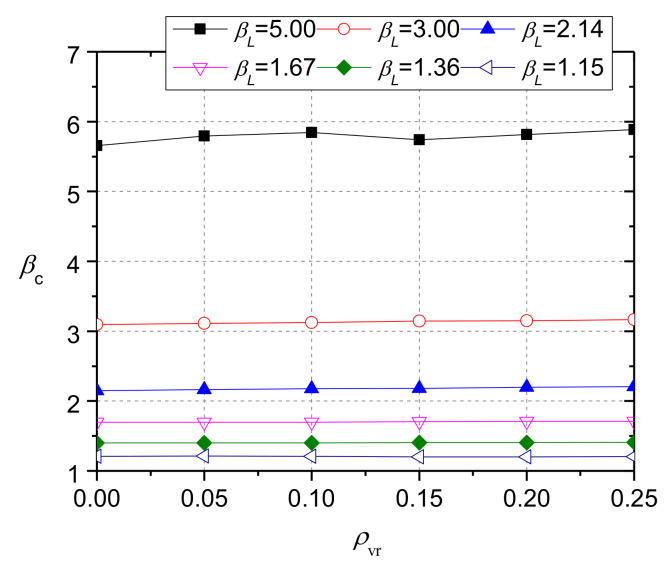
Effect of rubber content on the value of *β*_c_ when concrete compressive strength is 25 MPa.

Figure 22 shows the calculation results of concrete with the same compressive strength of 25 MPa, but different rubber contents. Although the calculation results of *β*_c_ fluctuated with *ρ*_vr_ when *β*_L_ was 5.0, the influence of rubber concrete on *β*_c_ was very limited. This indicates that, when the compressive strength of CRC is determined, its bearing strength is not affected by the rubber contents. Thus, the reason the results of the three groups of specimens in the experiment differed slightly is that their difference in compressive strength was small.

**Figure 23:**
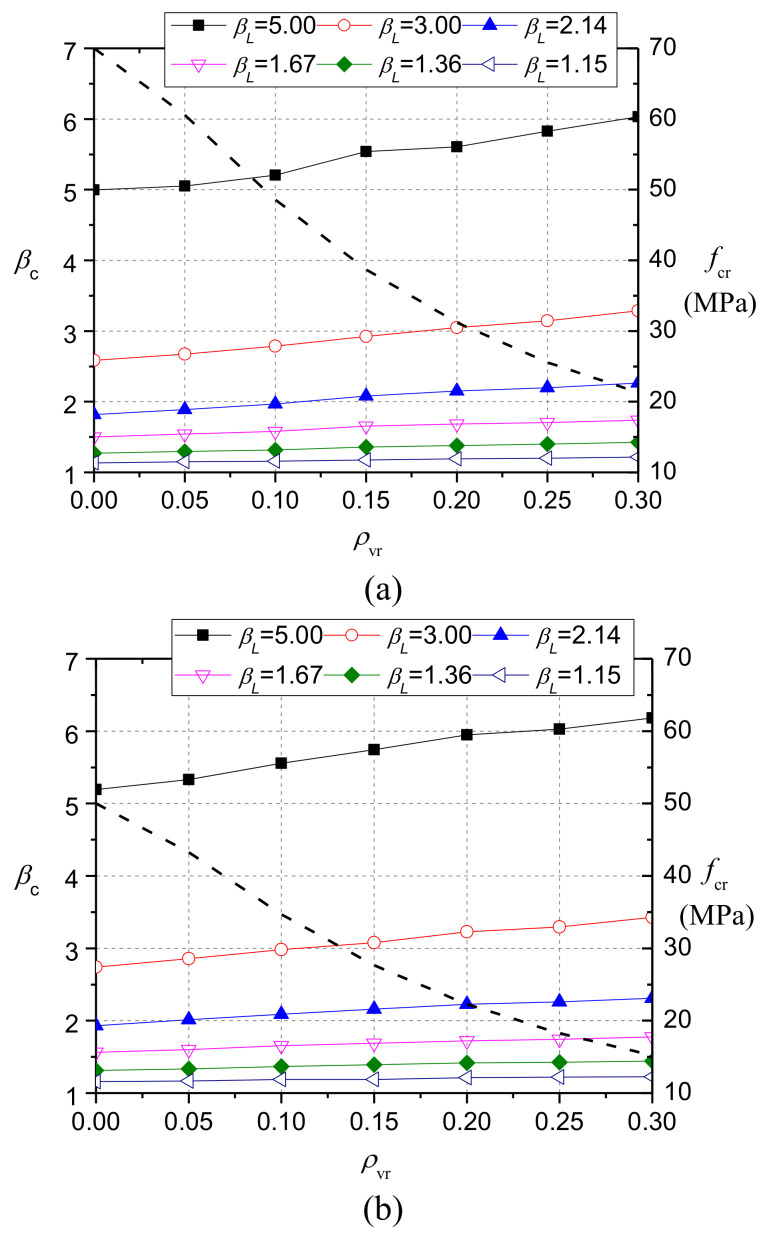
Effect of rubber content on the value of *β*_c_ when the compressive strength of reference NC is the same: (**a**) 70 MPa; (**b**) 50 MPa.

Further discussions were made by calculating bearing strength of CRC with different compressive strength. Figure 23a,b show the calculation results of concrete with different rubber contents when the compressive strengths of the reference NC were 70 MPa and 50 MPa, respectively. The dashed lines in the figures are the compressive strength of CRC. With the increase of rubber content and the decrease of the axial compressive strength, *β*_c_ increased notably.

## 6. Discussion

The above numerical results indicated that the compressive strength of CRC is the main factor affecting its bearing strength. In fact, Roberts-Wollmann et al. [28] pointed out that the ratio (*f*_ct_/*f*_c_) is one of the key factors determining the bearing strength. A new equation including the ratio (*f*_ct_/*f*_c_) as a parameter was proposed for the prediction of the bearing strength based on a Mohr failure criterion [28]:

(21) $$F_{u}=\frac{f_{c}A_{l}}{\beta \left(f_{c}/f_{\mathrm{ct}}\right)+\alpha }$$

where the parameters *α* and *β* depend on the dimensions of the loading plate and concrete block. The fact that lightweight concrete specimens exhibited significantly lower bearing strengths than normal-weight concrete specimens was found to be because lightweight concrete has a lower value of ratio (*f*_ct_/*f*_c_) [28]. The significant influence of this ratio on the bearing strength can be also derived from the experimental results reported by Zhao [37], who completed axial compression tests on four groups of 48 reactive powder concrete (RPC) prism specimens. The test results show that, with the addition of steel fibers, the increase in the tensile strength of concrete was more notable than that in compressive strength. As a result, the ratio (*f*_ct_/*f*_c_) increased in RPC with steel fibers and *β*_c_ was larger than that of RPC without steel fibers.

**Figure 24:**
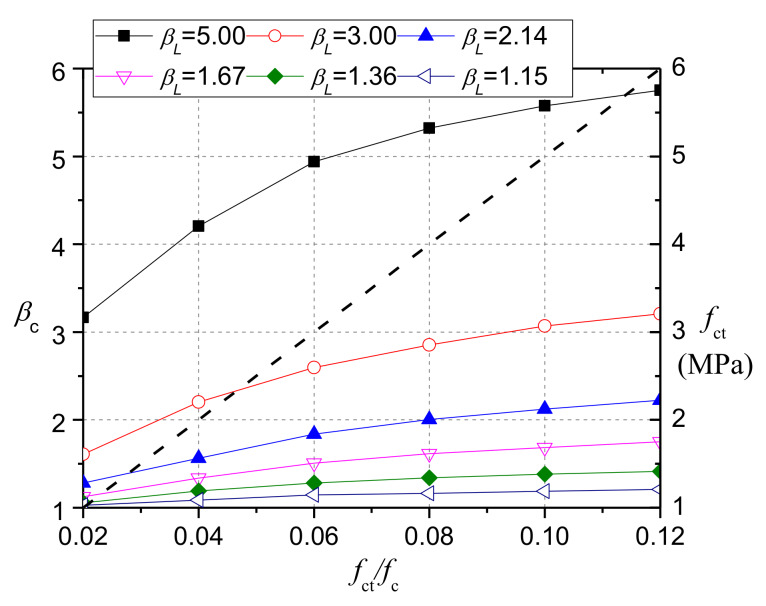
Effect of the ratio (*f*_ct_/*f*_c_) on the value of *β*_c_.

Figure 24 shows the calculation results for concrete with different tensile strength, while the compressive strength is 50.0 MPa. Obviously, *β*_c_ increases as the ratio (*f*_ct_/*f*_c_) increases. However, for CRC of the same compressive strength with NC, the influence of rubber contents on the ratio (*f*_ct_/*f*_c_) is small [8], and the tensile strength (*f*_ct_) of both CRC and NC was calculated according to Equation (16). As a result, the rubber content does not have an important effect on the bearing strength of CRC, which has been confirmed by experimental results. However, when the compressive strength of CRC is smaller than that of NC, the ratio (*f*_ct_/*f*_c_) is larger, as indicated by Equation (22), which was obtained by dividing both sides of Equation (16) by *f*_cr_. Then, the bearing strength of CRC would be larger than that of NC.

(22) $$\frac{f_{\mathrm{ctr}}}{f_{\mathrm{cr}}}=\frac{0.26}{f_{\mathrm{cr}}^{1/3}}$$

## 7. Conclusions

In this paper, the bearing strength of CRC under partial area loading was investigated via experimental and numerical analysis. The main conclusions can be drawn as follows.

- From experimental observation, the CRC and NC specimens show similar failure modes and deformation properties, which implies that rubber particles have not changed the force transfer and failure mechanism of concrete under partial area loading. At the same time, the measurement results of failure wedge indicate that the difference in the dilation angle of the two types of concrete is not as large as expected.
- Although rubber particles have an effect on some mechanical properties of concrete such as elastic modulus, Poisson’s ratio, and dilation angle, it can be seen through finite element parameter analysis that these parameters have little influence on the bearing strength of concrete. On the other hand, CRC and NC have a small difference in the key mechanical properties that affect bearing strength, such as properties under multi-axial compression, the ratio of tensile strength to compressive strength, and so on. This ultimately leads to the similar bear strength of CRC and NC when their compressive strength is the same.
- The existing formula for calculating the bearing strength of NC can be used to predict that of CRC, including its value at early concrete age, which is very beneficial to the design of CRC structural members.

The results in this study would alleviate the concerns of civil engineers on calculating the bearing strength of CRC. The important factors that affect the bearing strength of concrete were revealed. It is suggested to study the bearing strength of steel-fiber reinforced CRC to further evaluate the effect of the ratio of tensile strength to compressive strength on the bearing strength.

## Figures and Tables

**Table 1:** Mix proportions of the concrete (kg/m^3^). NC, normal concrete; CRC, crumb rubber concrete.

Group	Rubber Replacement Ratio	Rubber	Cement	Coarse Aggregate	Sand	Water	Water Reducer	Age /Days
NC	0	0	298	1297	610	168	1.49	28
CRC5E	5.0%	100	362	1297	556	168	1.81	14
CRC5	5.0%	100	362	1297	556	168	1.81	28
CRC10	10.1%	200	598	1240	414	168	3.00	28

**Table 2:** Compressive strength and bearing strength of concrete (MPa).

Side Length of Loading Plate		150 mm				75 mm				50 mm			
Specimen Number		1	2	3	Mean	1	2	3	Mean	1	2	3	Mean
Group	NC	29.3	28.4	×	28.9	56.7	56.4	49.4	54.2	93.6	64.0 *	91.8	92.7
	CRC5E	18.0	18.4	×	18.2	42.1	41.4	39.3	40.9	62.0	60.4	63.2	61.9
	CRC5	22.5	21.7	21.1	21.8	45.0	40.9	42.5	42.8	67.6	66.8	61.6	65.3
	CRC10	20.4	19.4	21.4	20.4	37.9	39.1	30.2	35.7	54.2	68.8	58.6	60.5

Note: Symbol × for an unsuccessfully tested specimen; symbol * for the unused data, which is 30% larger or smaller than the mean of other data in the group.

**Table 3:** Carbon intensities of ingredients of various concretes.

Ingredients	Cement	Coarse Aggregate	Sand	Waste Rubber, Grinding	Water Reducer	Waste Rubber, Disposal
CO_2_ emissions, kg-CO_2_/kg	0.98	0.00312	0.045	0.62	1.22	3.18

**Table 4:** CO_2_ emission of concrete (kg/m^3^).

Mixtures	NC	CRC5	CRC10
CO_2_ emission	325.35	130.03	100.20

**Table 5:** Summary of the geometry of failure wedges.

Specimens	NC-75-2	NC -50-1	CRC5-75-1	CRC5-50-3	CRC10-75-2	CRC10-50-3
*a* (mm)	75	50	75	50	75	50
*h* (mm)	91	87	116	71	74	71
*θ*	22.4°	16.0°	17.9°	19.4°	26.9°	19.4°
*φ*	45.2°	57.9°	54.2°	51.2°	36.3°	51.2°
